# Cell Polarity in Cerebral Cortex Development—Cellular Architecture Shaped by Biochemical Networks

**DOI:** 10.3389/fncel.2017.00176

**Published:** 2017-06-28

**Authors:** Andi H. Hansen, Christian Duellberg, Christine Mieck, Martin Loose, Simon Hippenmeyer

**Affiliations:** Institute of Science and Technology AustriaKlosterneuburg, Austria

**Keywords:** cerebral cortex, polarity, neurogenesis, neuronal migration, axon, dendrite, break in symmetry, GTPases

## Abstract

The human cerebral cortex is the seat of our cognitive abilities and composed of an extraordinary number of neurons, organized in six distinct layers. The establishment of specific morphological and physiological features in individual neurons needs to be regulated with high precision. Impairments in the sequential developmental programs instructing corticogenesis lead to alterations in the cortical cytoarchitecture which is thought to represent the major underlying cause for several neurological disorders including neurodevelopmental and psychiatric diseases. In this review article we discuss the role of cell polarity at sequential stages during cortex development. We first provide an overview of morphological cell polarity features in cortical neural stem cells and newly-born postmitotic neurons. We then synthesize a conceptual molecular and biochemical framework how cell polarity is established at the cellular level through a break in symmetry in nascent cortical projection neurons. Lastly we provide a perspective how the molecular mechanisms applying to single cells could be probed and integrated in an *in vivo* and tissue-wide context.

## Establishment of Cellular Polarity in Sequential Stages of Cortical Development

### Neural Stem Cell Polarity

**Figure 1:**
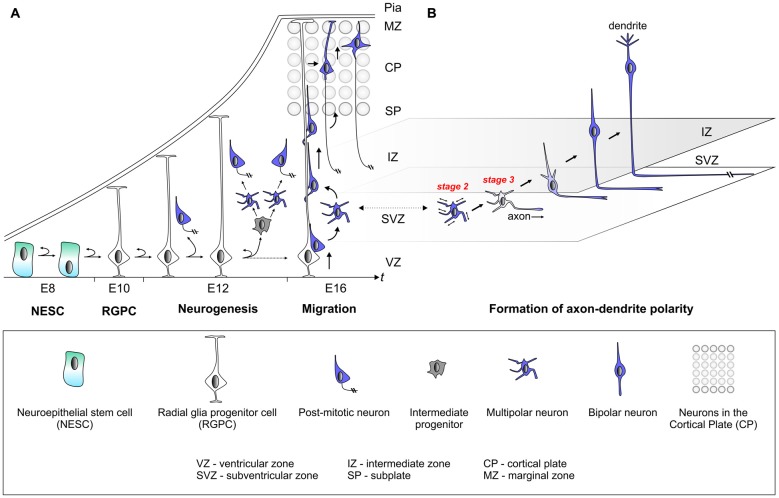
Establishment of cell polarity in cerebral cortex development. **(A)** The early neuroepithelium is composed of highly polarized neuroepithelial stem cells (NESCs, apical-basal polarity is indicated). NESCs give rise to radial glia progenitor stem cells (RGPCs) which exhibit even more polarized cellular morphology with an extended basal process. During neurogenesis symmetric radial glia progenitor (RGP) divisions may generate two RGPs but asymmetric divisions produce a renewing RGP and a neuron or an intermediate progenitor (IP). IPs further divide symmetrically in the subventricular zone (SVZ) to produce neurons. The basal processes of RGPs serve as a scaffold for nascent post-mitotic neurons, which migrate in a step-wise fashion coupled with changes in cell polarity, from the ventricular zone (VZ)/SVZ through the intermediate zone (IZ) in order to reach the cortical plate (CP). After nascent cortical projection neurons have delaminated from the neuroepithelium at the ventricular surface they move radially away to the SVZ exhibiting bipolar (BP) morphology. Within the SVZ/IZ, neurons “sojourn” for about 24 h or longer and most adopt a multipolar (MP) morphology, extending and retracting processes in all directions. At one point fundamental cellular polarization events take place that predetermine the future axon of the neuron before the neuron again adopts a bipolar morphology and starts locomoting along the radial glial fiber through the IZ. Once reaching the subplate (SP), neurons enter the CP and migrate towards the marginal zone (MZ) where they detach from the radial glial fiber. Finally, neurons settle in their appropriate position in the CP and the leading process will eventually become the dendrite. **(B)** This panel depicts the migrating neuron from panel **(A)** in higher detail with the leading and trailing processes which eventually become the dendrite and axon, respectively.

The mammalian cerebral cortex emerges from the neuroectoderm. At the end of neurulation and neural tube closure, occurring from embryonic day (E) 7 to E9 in mice, the early neuroepithelium is composed of neuroepithelial stem cells (NESCs) from which all subsequent neural progenitor cells and their neuron lineages derive (Figure 1). NESCs are highly polarized and their nuclei exhibit interkinetic nuclear migration whereby they translocate from the ventricular (apical) side to the more basal side in concert with the cell cycle (Lee and Norden, 2013). NESC polarity correlates with the asymmetric distribution of cell fate determinants which are thought to control the fine balance between symmetric and asymmetric progenitor divisions (Shitamukai and Matsuzaki, 2012). Such balance is critical for the generation of the correct number of radial glia progenitor cells (RGPCs), which are not only lineally related to NESCs but exhibit even more polarized cellular morphology with an extended basal process (Taverna et al., 2014). In the initial stages of neurogenesis, NESCs arrange the mitotic spindle in parallel (division plane perpendicular) to the ventricular zone (VZ) and divide mostly symmetrically, thereby expanding the progenitor pool (Postiglione and Hippenmeyer, 2014; Taverna et al., 2014). The disruption of the mitotic spindle, anchored to the lateral walls of NESCs, results in the precocious generation of neurons and apoptosis (Yingling et al., 2008). Thus the correct cellular polarization of the earliest neural progenitor cells in the developing cerebral cortex is absolutely essential for the correct lineage progression and eventual neuron production. While it has been well established that components of the planar cell polarity signaling pathway play critical roles in establishing and maintaining progenitor polarity (Knoblich, 2008; Homem et al., 2015), the signaling cues and molecular mechanisms that instruct polarization and the break of symmetry in NESCs are not well understood *in vivo*.

Radial glia progenitors (RGPs) have been demonstrated to be the major neural progenitors in the developing cortex responsible for producing the vast majority of cortical excitatory neurons (Malatesta et al., 2000; Noctor et al., 2001; Anthony et al., 2004; Lui et al., 2011; Franco and Muller, 2013; Borrell and Götz, 2014; Taverna et al., 2014). The RGP division patterns and dynamics determine the number of neurons in the mature cortex. RGP cell division during mitosis occurs at the surface of the embryonic VZ and can be either symmetric or asymmetric, which is defined by the fate of the two daughter cells (Lui et al., 2011; Gao et al., 2014; Taverna et al., 2014; Homem et al., 2015). The extrinsic and intracellular signaling cues that instruct the mode of cell division are not well understood. The directional segregation of cell fate determinants such as Notch, components of the planar cell polarity signaling module, or entire centrosomes (i.e., duplicated centrioles) in dividing neural stem cells indicates however that polarized secretion and/or trafficking is a key mechanism (Wang et al., 2009; Lui et al., 2011; Paridaen et al., 2013; Taverna et al., 2014). Symmetric RGP divisions generate two RGPs to amplify the progenitor pool or two postmitotic neurons. In contrast, asymmetric divisions produce a renewing RGP and a neuron or an intermediate progenitor (IP). IPs can further divide symmetrically in the subventricular zone (SVZ) to produce neurons (Noctor et al., 2004; Kowalczyk et al., 2009). Interestingly, IPs adopt a multipolar morphology (Noctor et al., 2004; Kowalczyk et al., 2009) and it is currently not known whether the transition from bipolar (RGP) to multipolar (IP) state could correlate with, or even be instructive, for the neurogenic potential in dividing IPs. RGPs may also produce other types of transient amplifying progenitors, such as short neural precursors (SNPs; Stancik et al., 2010) and outer SVZ radial glial progenitors (oRGs aka basal RGs or bRGs; Fietz et al., 2010; Hansen et al., 2010; Shitamukai et al., 2011; Wang X. et al., 2011; Kelava et al., 2012; Betizeau et al., 2013; Florio et al., 2015; Johnson et al., 2015; Pollen et al., 2015). Although oRGs like RGPs are bipolar, they have been shown to adopt different morphological states and thus likely exhibit distinct cellular polarity since they lack apical attachment at the ventricle. Distinct oRG morphologies may reflect distinct competence states with respect to the number and types of neurons which are generated (Betizeau et al., 2013). Although the above studies provide a framework of stem cell polarity and lineage progression at the cellular level (Figure 1), the underlying molecular and biochemical mechanisms of progenitor cell polarization are still poorly defined.

### Polarity in Nascent Postmitotic Neurons—Implications for Neuronal Migration

The basal processes of RGPs serve as a scaffold for nascent cortical neurons, which migrate from the VZ/SVZ through the intermediate zone (IZ), in order to reach the cortical plate (CP; Rakic, 1972; Evsyukova et al., 2013). Cortical layering occurs in an “inside-out” fashion whereby earlier born neurons populate deep layers and later born neurons occupy progressively upper layers (Angevine and Sidman, 1961; Rakic, 1974). Newly-born cortical neurons migrate, in a step-wise fashion coupled with changes in cell polarity, from the VZ/SVZ through IZ zone in order to reach the CP where they position themselves at their final location (Figure 1; Rakic, 1972; Nadarajah and Parnavelas, 2002; Noctor et al., 2004; Tsai et al., 2005; Marín et al., 2010; Hippenmeyer, 2014). Timelapse and videomicroscopy approaches (Tabata and Nakajima, 2008; Noctor, 2011; Tsai and Vallee, 2011) with the goal to trace the migration paths of individual cortical projection neurons have impressively revealed that: (1) radially migrating neurons proceed though several distinct migratory phases; (2) change their morphology and polarize along the way; and (3) adjust their mode of migration while transiting through the different zones along the radial migratory path (Nadarajah et al., 2001; Tabata and Nakajima, 2003; Noctor et al., 2004; Tsai et al., 2005; Sekine et al., 2011; Figure 1). From these observations through live-imaging, it is evident that nascent migrating neurons undergo a series of morphological changes including the depolarization and repolarization within the SVZ/IZ. The molecular mechanisms controlling these morphological transitions are poorly defined but if they are perturbed or delayed, the development of the cortical cytoarchitecture may be compromised. This is in particular relevant in humans that suffer from e.g., Lissencephaly (a severe cortical malformation disorder) where the loss of *LIS1* activity results in a defect to repolarize migrating neurons which in turn accumulate in ectopic positions instead of properly migrating into the developing CP (Tsai et al., 2005; Wynshaw-Boris et al., 2010). LIS1 is only one of many molecules which are involved in more than one cellular polarization process. As such LIS1 plays a role in neural progenitor polarization and in the establishment of polarity in postmitotic neurons. It will thus be important to precisely dissect the sequential and/or distinct functions of proteins orchestrating cellular polarity during development.

### Establishment of Axon and Dendrite Compartments in Cortical Projection Neurons

After nascent cortical projection neurons, exhibiting bipolar (BP) morphology, have delaminated from the neuroepithelium at the ventricular surface they move radially away to the SVZ. Within the SVZ neurons “sojourn” for about 24 h or longer and most adopt a multipolar (MP) morphology, extending and retracting processes in all directions (Tabata and Nakajima, 2003; Noctor et al., 2004). While this stage is critical for the progression of the sequential migration program it is also essential for establishing the cellular compartments that later transform into axonal and dendritic processes. During this phase, multipolar (MP) neurons tend to migrate tangentially in an apparent random fashion (Noctor et al., 2004; Jossin and Cooper, 2011). At one point however, fundamental cellular polarization events take place that predetermine the future axon of the neuron (Barnes and Polleux, 2009) before the neuron again adopts a bipolar morphology (Figure 1). In the remainder of this review we synthesize a framework of neuronal polarization based upon *in vitro* biochemical, cell culture and genetic loss of function experiments *in vivo*. We reflect upon the relative contribution of extrinsic cues and cell-intrinsic molecular and biochemical signaling modules that dictate the break in symmetry and control polarization of cortical projection neurons.

## Extracellular Cues Controlling Projection Neuron Polarity in Cortex Development

**Figure 2:**
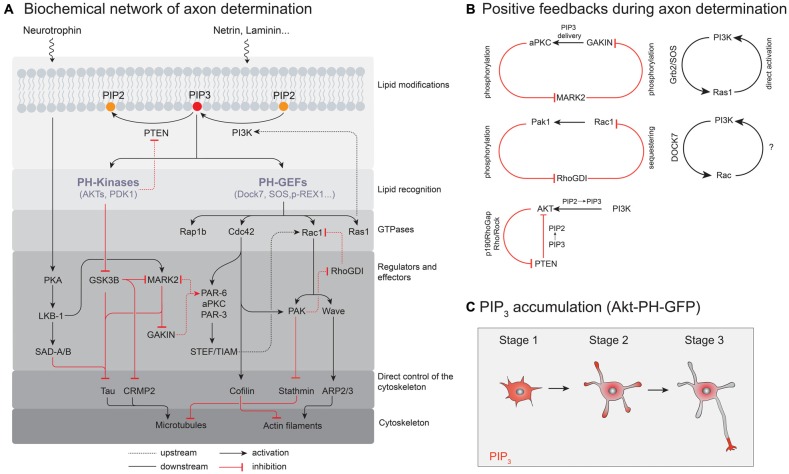
Molecular signaling pathways controlling neuronal polarization. **(A)** A simplified illustration of the biochemical network of axon determination. Only interactions localized to the nascent axon are shown. **(B)** Positive feedback loops in the process of axon determination. **(C)** Probing PIP_3_ localization and accumulation in polarizing neurons with Akt-pleckstrin-homology (PH)-GFP as a probe for PIP_3_.

**Figure 3:**
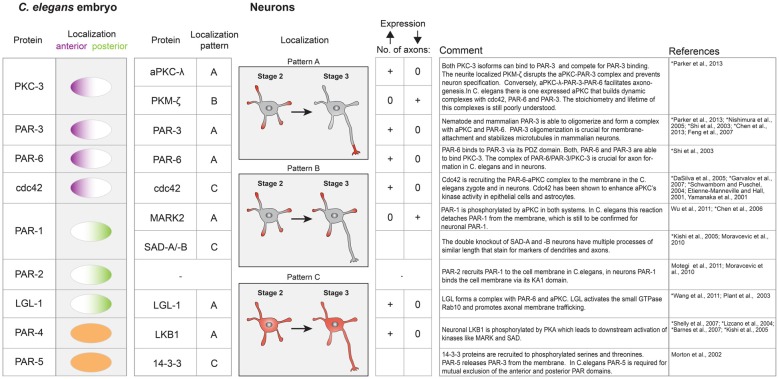
Table of key polarity proteins in *C. elegans* and their neuronal homologs. The localization of the nematode proteins is illustrated according to their anterior or posterior domain affiliation. In neurons the respective localization is classified according to the indicated patterns (A–C). A supernumerary axon phenotype is indicated by a plus sign, while 0 represents the absence of an axon upon overexpression or downregulation of the respective polarity protein. References describing neuronal protein systems are marked with an asterisk.

Developing cortical neurons can break symmetry in the absence of external cues suggesting that the role of the extracellular signals in the *in vivo* context is solely to activate/trigger an intrinsic symmetry-breaking pathway. The intrinsic signaling pathways on the other hand are dependent on the internal biochemical state of the cell (Figures 2, 3 and see below for detailed discussion). Albeit cell intrinsic mechanisms have received much more attention than extracellular regulatory cues it is clear that in the developing cortex, cell-to-cell interactions, the local microenvironment and long-range signaling constitute essential factors for the establishment of projection neuron polarity *in vivo*.

### Cell-Cell Interactions

Nascent projection neurons are embedded in a heterogeneous environment and cell-cell interactions are likely to play an important role in neuronal polarization (Jossin, 2011; Gartner et al., 2015; Namba et al., 2015). It has been suggested that the radial glial scaffold, on which neurons perform locomotion in the IZ, could be involved in the MP-to-BP transition. Experiments inhibiting the cell-adhesion molecule N-cadherin have shown that newly-born neurons expressing a dominant-negative form of N-cadherin establish abnormal leading processes (Gartner et al., 2012, 2015). These experiments have also indicated that radial glial-neuron interactions mediated by N-cadherin play an essential role in the initial radial alignment of nascent neurons and thus possibly (albeit in an indirect manner) in the subsequent MP-to-BP transition. Interestingly, polarized N-cadherin localization has been shown to occur in a single neurite during MP-to-BP transition and thus likely represents one of the earliest consequences of the symmetry-break (Gartner et al., 2012). In such context, it has been proposed that the interaction of multipolar cells and RGPs mediated by N-cadherin leads to the establishment of axon-dendrite polarity through polarized distribution of active RhoA in the neurite contacting the RGC and active Rac1 on the opposite side where the axon is formed (Xu et al., 2015). Physical interactions between pioneering axons from earlier generated neurons and the dynamic neurites from newly born neurons have been shown to contribute to polarization in MP neurons (Namba et al., 2014, 2015). These interactions involve the cell adhesion molecule transient axonal glycoprotein 1 (TAG-1). The highest expression of TAG-1 has been observed in the lower IZ (Namba et al., 2014), exactly where nascent neurons switch from MP-to-BP morphology. Current models propose that TAG-1 is expressed in both MP cells and pioneering axons and thus could mediate homophilic cell-cell contacts. Indeed, shRNA-mediated knockdown of TAG-1 results in the disruption of the MP-to-BP transition and axon specification. The underlying mechanism of TAG-1 action in polarization may involve: (1) an increase in physical tension in the immature neurite leading to axon induction and formation; and (2) contact-mediated activation of signaling molecules that instruct axon specification (Namba et al., 2015). Interestingly, N-cadherin is mainly expressed in the upper IZ (Xu et al., 2015) but TAG-1 in the lower IZ (Namba et al., 2014). Thus N-cadherin and TAG-1 could act as two separate polarity inducing cues which might work complementary in axon-dendrite formation as proposed by the Kaibuchi laboratory (Xu et al., 2015). Whether the induction of cellular polarization within these two distinct zones correlates with a certain neuron type (e.g., derived from either RGCs or IPs) remains to be determined.

### Secreted Factors

#### Reelin

Newly born cortical projection neurons migrate from the VZ to the CP in order to reach their final target area (Marín et al., 2010; Hippenmeyer, 2014). A key regulatory module controlling neuronal migration includes the Reelin pathway (Honda et al., 2011). The function of Reelin in neuronal migration has been studied extensively for decades and several hypotheses concerning the mechanism of Reelin action have been put forward (Honda et al., 2011). However, it has also become clear recently that Reelin fulfills an important role in the polarization of nascent projection neurons (Jossin and Cooper, 2011; Jossin, 2011). Reelin is mainly expressed by Cajal-Retzius cells in the marginal one (MZ) in the developing cortex (Ogawa et al., 1995). The Reelin protein primarily binds to its two cognate receptors, very low density lipoprotein receptor (VLDLR) and apolipoprotein E receptor 2 (ApoER2/LRP8; D’Arcangelo et al., 1999), which are mainly expressed in RGPs and nascent migrating neurons (Perez-Garcia et al., 2004). Binding of Reelin to its receptors triggers the activation of a Src family kinase (SFK) called Fyn which in turn phosphorylates the adaptor protein disabled-1 (DAB1; Howell et al., 1997, 1999). Phosphorylated DAB1 functions as a hub for several downstream intracellular signals and has been shown to activate the effectors CRK, C3G and PI3K which in turn regulate the activity of Limk1, Akt and Rap1 to eventually modulate the dynamic cytoskeleton (Honda et al., 2011; Sekine et al., 2014). Thus the Reelin-DAB1 pathway translates extracellular cues into cytoskeletal changes in migrating neurons (Frotscher, 2010; Forster et al., 2010). How Reelin might regulate dynamic polarization events in nascent cortical projection neurons is less well understood. Interestingly however, it has been shown that while VLDLR is mainly localized on the leading processes of migrating neurons in the MZ, ApoER2 is primarily localized to neuronal processes and the cell membranes of multipolar neurons in the SVZ and lower IZ. In addition to strong expression of Reelin in the MZ, it was also demonstrated that Reelin is also expressed in the IZ at early developmental stages (Hirota et al., 2015). *Ex vivo* experiments where exogenous Reelin was added to cultured brain slices have shown an effect on the morphology and dynamic behavior of nascent neurons in the IZ (Britto et al., 2014). Thus, based on the expression pattern of Reelin and its cognate receptors it is conceivable that Reelin could play a prominent role during the polarization process of nascent cortical projection neurons. Indeed, Jossin and Cooper propose a three step model (Jossin and Cooper, 2011) how Reelin controls the radial orientation of multipolar neurons in SVZ/IZ. First, multipolar neurons migrate tangentially in a stochastic manner in the SVZ/IZ until they encounter Reelin which leads to the activation of the small GTPase RAP1, likely via pDAB1-CRK/CRKL-C3G signaling (Ballif et al., 2004; Voss et al., 2008). Next, active RAP1 triggers an increase of the surface level of N-Cadherin in multipolar neurons. These increased cell surface levels of N-Cadherin could then allow the multipolar neurons to sample local microenvironmental cues which then could initiate the break in symmetry and induce polarization. The cortical projection neurons then progressively exit the multipolar stage and adopt a bipolar morphology (Jossin, 2011; Jossin and Cooper, 2011). Altogether, the above data and model indicates that Reelin acts as a critical cue for the directional movement of nascent migrating cortical projection neurons and could serve as a critical extracellular cue for modulating polarization of nascent migrating cortical projection neurons. It will be intriguing to decipher the precise intracellular and biochemical signaling pathways controlling RAP1-dependent N-Cadherin trafficking and how N-Cadherin-dependent signaling triggers the break in symmetry.

#### Neurotrophins

Brain derived neurotrophic factor (BDNF) and neurotrophin-3 (NT-3) are highly expressed in the developing brain and have been shown to stimulate axon specification and elongation (Morfini et al., 1994; Nakamuta et al., 2011). Both, BDNF and NT-3 as extracellular regulators of neuronal polarity are of special interest since they act in an autocrine and/or paracrine manner in cell-culture (Nakamuta et al., 2011). This feature indicates that neurons are able to produce extracellular stimuli (in form of secreted neurotrophins) that activate the intrinsic machinery for axon-dendrite specification in a cell-autonomous and non-cell-autonomous manner. BDNF and NT-3 bind to tropomyosin related kinases receptors (TRK), TRK-B and TRK-C respectively (Chao, 2003). Upon TRK receptor binding, the small GTPase Ras and PI3K are activated. This leads to the production of phosphatidylinositol (3,4,5)-trisphosphate (PIP_3_) and the activation of its downstream signaling pathways (Reichardt, 2006). Neurotrophin signaling through TRK receptors also leads to increased levels of inositol triphosphate (IP3)-induced calcium release which in turn activates the calmodulin-dependent protein kinase kinase (CaMKK) and calmodulin-dependent protein kinase I (CamKI; Nakamuta et al., 2011). The activation of CaMKK and CamKI triggers the phosphorylation of microtubule affinity regulating kinase 2 (MARK2). This leads to the phosphorylation of downstream microtubule associated proteins (MAPs) MAP2/4, Tau and DCX which reduces microtubule stability (Drewes et al., 1997; Schaar et al., 2004; Nakamuta et al., 2011). Interestingly, acute knockdown of MARK2 has been shown to stall MP-to-BP transition in the IZ in mice (Sapir et al., 2008). Thus, proper regulation of MARK2 appears to be essential for neuronal polarization *in vivo*.

BDNF signaling via TrkB has been demonstrated in culture experiments to lead to the activation of LKB1 (liver kinase b1 in mammals and PAR-4 in *C. elegans* (Figures 2, 3; Shelly et al., 2007). Loss of function of LKB1 either by genetic knockout or knockdown by shRNA in nascent cortical projection neurons results in striking phenotypes: axon specification is completely abolished while the dendrite appears to still be specified (Barnes et al., 2007; Shelly et al., 2007). In contrast, overexpression of LKB1 in neural progenitors and postmitotic neurons lead to formation of multiple axons. In a biochemical pathway downstream of BDNF/TrkB, LKB1 is phosphorylated by protein kinase A (PKA) or ribosomal S6 kinase (p90RSK) at Serine 431 (Collins et al., 2000; Sapkota et al., 2001). Phosphorylated LKB1 leads to downstream activation of MARK2 (Shelly et al., 2007) and the SAD kinases which in turn phosphorylate Tau-1 (Kishi et al., 2005; Barnes et al., 2007). Remarkably, SAD-A/B double knockout precisely mimic the LKB1 loss of function phenotype with complete absence of the axon (Kishi et al., 2005). In summary, the above studies established a model whereby BDNF signaling via TrkB results in the activation of LKB1 which is translated into an intracellular symmetry break in multipolar cortical projection neurons while sojourning in the SVZ/IZ. Phosphorylated LKB1 localizes into the nascent axon and is required for axon extension and development. It will be interesting to determine the extent of specificity and functional redundancy of individual downstream components along the BDNF/TrkB-LKB1-SAD-A/B signaling module while executing the break in symmetry.

#### Semaphorins

Semaphorins consist of a large family of membrane bound or secreted proteins (Nakamura et al., 2000). The secreted semaphorin, Sema3A have been shown to act as a chemotactic factor for migrating cortical projection neurons (Polleux et al., 2000; Chen et al., 2008). Sema3A expression is highest near the pial surface in the developing cortex and the Sema3A expression domain establishes a descending gradient across the emerging cortical layers (Polleux et al., 2000; Chen et al., 2008). Sema3A binds its co-receptors Plexin and Neuropilin (Negishi et al., 2005) and it has been suggested that Sema3A may actively control the process of symmetry breaking and cellular polarization. Sema3A activates a number of downstream cascades resulting in the tuning of relative levels of cGMP and cyclic adenosine 3′,5′-monophosphate (cAMP) which negatively affects axon formation by downregulation of PKA-dependent phosphorylation of LKB1 (Shelly et al., 2011). Interestingly, exposure of undifferentiated neurites to local sources of Sema3A in hippocampal neuron cell culture leads to the suppression of axon-formation but promotion of dendrite formation in culture conditions (Shelly et al., 2011). Strikingly, *in vivo* knockdown of the Sema3A receptor neuropilin-1 in rat embryonic cortical progenitors results in severe polarization defects. Furthermore, Sema4D inactivates Ras (Oinuma et al., 2004) while it activates RhoA (Swiercz et al., 2002) which prevents axon formation and/or outgrowth via reduced actin dynamics and actin contraction. Thus, Sema3A acting via the neuropilin-1 receptor and semaphorins in general are critically involved in the symmetry break and polarization of nascent projection neurons in the developing cortex (Shelly et al., 2011).

#### TGF-β

The transforming growth factor β (TGF-β) has been reported to play an important role in the polarization of nascent cortical projection neurons in the developing cerebral cortex (Yi et al., 2010). Upon binding of one of the three TGF-β ligands (TGF-β1-3) to the type II TGF-β receptor (TβR2), this receptor is recruited to the type I TGF-β receptor (TβR1) to form a complex which triggers the phosphorylation of the two receptors by the serine/threonine kinase domain (Shi and Massagué, 2003). The TβR2-TβR1 receptor complex has been shown to phosphorylate Par-6 which in turn regulates Cdc42/Rac1 activity by recruiting the ubiquitin ligase Smurf1 which promotes proteasomal degradation of the RhoA GTPase. This results in reduced activity of RhoA in the nascent axon thereby stimulating its outgrowth (Gonzalez-Billault et al., 2012). Thus, RhoA activity can be precisely regulated in response to TGF-β signaling thereby controlling the dynamics of the local actin organization which is essential for axon specification and thus cellular polarization (Yi et al., 2010). Interestingly, TGF-β2-3 is highly expressed near the VZ/SVZ. Thus nascent developing neurons could be exposed to a gradient which delivers a uniform stimuli for axon specification (Yi et al., 2010). However, the majority of MP neurons extend their axons tangentially (Hatanaka and Yamauchi, 2013) rather than towards the ventricular side. It is thus conceivable that TGF-β might only act as a stimulus for axon specification rather than an axon guidance cue (Yi et al., 2010). Bone morphogenic protein (BMP), also a member of the TGF-β superfamily appears to play important functions in the MP-to-BP transition as well. BMPs are known to signal via the intracellular downstream mediator SMAD which leads to the suppression of collapsin response mediator protein 2 (CRMP2), a transcription factor known to promote microtubule assembly (Shi and Massagué, 2003; Sun et al., 2010). Strikingly, upon suppression of CRMP2 or overexpression of dominant negative forms of CRMP2 multipolar cells accumulate in the SVZ/IZ in the developing cortex. While these findings suggest that a BMP-SMAD signaling pathway, via CRMP2, regulates the polarization of cortical projection neurons the precise molecular and biochemical mechanisms remain to be determined (Sun et al., 2010). Altogether, different members of the TFG-β superfamily play important roles in multipolar cortical neurons and direct neuronal polarization through distinct signaling pathways.

## Intrinsic Biochemical Networks That Mediate Neuronal Polarity

While the above sections illustrated the role of extracellular cues for triggering and/or execution of neuronal polarization, the intrinsic molecular mechanisms involved in symmetry breaking will be discussed in the sections below with a focus on *in vitro* and cell culture experiments. Isolated neurons in cell culture form one axon and several dendrites. External chemical or physical cues of instructive or antagonistic nature that determine axon formation have been identified (Lamoureux et al., 2002; Gomez et al., 2007; Shelly et al., 2011). However, cultured neuronal cells are able to polarize even in the absence of any external cue (Dotti et al., 1988) suggesting that cells have intrinsic ability to break symmetry, which is solely activated externally. What are the functional cell-intrinsic networks that underlie cell polarization and determine the biochemical state of the cell? Based on Turing’s idea of a reaction-diffusion mechanism to explain how spatial order during embryogenesis may arise (Turing, 1990), Gierer and Meinhardt developed a conceptual framework for pattern formation, which is based on the local activation in the form of self-enhancing feedback, which amplifies and reinforces spatially asymmetric distributions of molecules, coupled to long-range inhibitory processes (Meinhardt and Gierer, 2000). While this concept was originally developed to explain spatial patterning during morphogenesis, it also provides a framework to understand cell polarity. Accordingly, cell polarization is seen as a self-organized process (Wennekamp et al., 2013), which involves local symmetry breaking, signal amplification and long-range inhibition (Wang, 2009; Chau et al., 2012).

Previous work was able to identify many molecular players involved in the processes that allow a neuron to choose the one neurite to become an axon (Barnes and Polleux, 2009). While cell polarization can theoretically arise from a single molecular species that features a positive feedback (Altschuler et al., 2008), symmetry breaking in neurons likely reflects interactions among multiple, partially redundant pathways with crosstalk among them (Namba et al., 2015). This network can be subdivided into several, partially overlapping modules, each of which comprises a subset of molecular players that encode for specific cellular functions. To a rough approximation, the output of one module can serve as the input for a module downstream. Here, we want to illustrate how these functional modules orchestrate neuronal polarization and how they are embedded in a more complex biochemical network giving rise to axon specification. Importantly, individual modules are often evolutionarily conserved among species and pathways that regulate cell polarization in seemingly distinct tissues and contexts are remarkably similar. Accordingly we can to some degree take advantage of known cell polarization concepts in yeast, *C. elegans* and migrating cells (Iden and Collard, 2008), with the goal to anticipate a better understanding of the molecular processes that underlie axon specification.

### PIP/PI_3_K Module

Molecules involved in the initial symmetry breaking event are commonly localized to the plasma membrane, where not only integral membrane proteins receive extracellular signals, but where also peripherally binding membrane proteins bind reversibly to the membrane (Cho and Stahelin, 2005). This restricts the diffusion of these proteins, increases the efficiency of protein-protein interactions and/or modulates their catalytic activity (Vaz et al., 1984; Leonard and Hurley, 2011; Ebner et al., 2017). As a result, the membrane can be interpreted as a computational platform where transient protein clusters integrate, interpret and amplify incoming biochemical signals (Groves and Kuriyan, 2010).

One component of membrane-based signaling pathways that was found to be essential for the establishment of intracellular organization include phosphoinositides (PIPs). Even though they represent only about 1% of membrane phospholipids (Di Paolo and De Camilli, 2006), the associated signaling pathways control cell growth, division, survival and differentiation, and allow to generate highly polarized neuronal morphologies such as growth cones and synapses (Sasaki et al., 2007). Cells use a precisely defined spatiotemporal distribution of PIPs to control the activity of intracellular signaling pathways. For cell polarity, it is the asymmetry of phosphatidylinositol-3,4,5-phosphate (PI(3,4,5)P_3_ = PIP_3_) at the plasma membrane that is used to establish a polarity axis in the cell. The intramembranous PIP_3_ concentration is controlled by activation of the phosphoinositide-3-kinase (PI3K; Whitman et al., 1985) as well as phosphatases such as phosphatase and tensin homolog (PTEN; Lee et al., 1999) that directly antagonize PI3K by dephosphorylating PIP_3_ to PI(4,5)P_2_ (PIP_2_) (Carracedo and Pandolfi, 2008). Overexpression of PTEN or inhibition of the PI3K were both found to abolish cell polarization and axon specification (Shi et al., 2003; Jiang et al., 2005). In contrast, reduction of PTEN expression results in neurons with multiple axons (Jiang et al., 2005). Together, these results demonstrate that the activities of these enzymes need to be tightly balanced to produce one and only one axon.

Since polarization is a dynamic spatiotemporal process, not only the total amount of phosphoinositides but also their distribution in space and time needs to be precisely regulated. In cells, the intracellular distribution of PIP_3_ can be visualized using a fluorescent reporter protein that specifically binds to PIP_3_; e.g., GFP fused to the Pleckstrin-homology (PH) domain of the serine/threonine kinase Akt (Gray et al., 1999). This probe visualized PIP_3_ accumulation at the tip of a neurite contributing to neuronal polarity and axon specification (Ménager et al., 2004). In contrast, EGFP-PLCd1-PH, which binds to PI(4, 5)P2 or IP3 showed a homogeneous distribution in cultured neuronal cells (Ménager et al., 2004).

The localized accumulation of PIP_3_ likely represents the first spatial landmark that establishes the polarity axes of the cell. *In vivo*, activity of PI3K and PIP_3_ production is most likely regulated by asymmetric distribution of extracellular factors (Namba et al., 2015), still, neurons polarize in cell culture without obvious asymmetries in their environment (Dotti et al., 1988). This suggest that starting from a homogeneous distribution of signaling molecules, dedicated biochemical circuits are able to amplify small fluctuations of signaling lipids in the plasma membrane. These interactions eventually break the symmetry of the cell and control cell morphogenesis (Wennekamp et al., 2013). The biochemical network underlying phosphoinositide polarity was studied in detail in other model systems. For example, *Dictyostelium discoideum* cells (Malchow et al., 1973), leukocytes and neutrophils (Trepat et al., 2012) polarize in response to a gradient of the chemoattractant cAMP or a variety of chemokines respectively by establishing domains of different phosphoinositides: PIP_3_ at the leading edge of the cell and PIP_2_ at its tail (Petrie et al., 2009). Importantly, and similar to neurons, the ability to break symmetry is independent from directional sensing, as cells that are placed in a uniform distribution of chemoattractant are still able to polarize (Petrie et al., 2009). Again, this illustrates the intrinsic ability of biochemical networks to polarize the cell in the absence of exogenous spatial signals (Wedlich-Soldner and Li, 2003). While phosphoinositide signaling was found to spatially organize the actin cytoskeleton, the initial symmetry breaking event itself does not depend on actin filaments. Fluorescently-labeled PH_Akt_ and PTEN, which acted as probes for PIP_3_ and PIP_2_ respectively, were found to self-organize into traveling waves in *Dictyostelium discoideum* cells even in the presence of the actin polymerization inhibitor latrunculin A (Gerisch et al., 2012). Importantly, this finding indicates that the ability to break symmetry in the membrane is established upstream and independent of the cytoskeleton. Instead, a PIP_3_-dependent negative regulation of PTEN recruitment to the membrane was suggested to allow PIP_3_ to accumulate. In addition, PTEN localization and activity has been found to be dependent on the small GTPase RhoA, which was found to restrict PTEN to the rear of chemotaxing leukocytes (Li et al., 2005) and *Dictyostelium* cells. Arai et al. (2010) further suggest a Ras-dependent positive feedback of PI3K activity to stabilize the polarized state of the cell. In addition, negative regulation of PTEN activity downstream of the PIP_3_ activated AKT kinase has been reported, which constitutes a parallel mechanism to maintain and stabilize polarity (Papakonstanti et al., 2007).

While phosphoinositides are the most important lipid species for cellular signaling there are also other lipid species involved in neuronal polarization: plasma membrane ganglioside sialidase (PMGS), which controls the ganglioside content in the plasma membrane of neurons was also found to show an asymmetric distribution of its activity: it is enriched in one of the stage 2 neurites and facilitates axon outgrowth by enhancing Rac and PI3K activity (Da Silva et al., 2005). Thus, the asymmetric distribution of two different kinds of lipid species appears to control the polarity of the cell.

### GEFs and Small GTPases

Which biochemical circuits underlie signal amplification in neurons? By now, the identity of several PI3K-dependent GTPases involved in neuronal polarization is known, such as H-Ras (Yoshimura et al., 2006b), Cdc42 (Garvalov et al., 2007) or Rap1B (Schwamborn and Püschel, 2004; Nakamura et al., 2013). Similar to PI3K (Shi et al., 2003) their overexpression results in supernumerary axons (see Figure 3), while their knock down prevents axon formation (Schwamborn and Püschel, 2004; Yoshimura et al., 2006b; Garvalov et al., 2007; Nakamura et al., 2013), however, these proteins do not interact with PIP_3_ themselves. Instead, the phosphorylation state of phosphoinositides in the plasma membrane is recognized by soluble guanine nucleotide exchange factors (GEFs) that contain PIP3 binding domains, such as GEFs of the Dbl and DOCK180 families (Rossman et al., 2005; Laurin and Côté, 2014). These protein families in turn activate GTPases while recruiting them to the membrane (Cherfils and Zeghouf, 2013). Though a systematic characterization of GEFs that directly interact with PIP_3_ and control cell polarization is not complete yet, candidate proteins include SOS and RasGFR, both members of the Dbl family of GEFs that contain a canonical DH-PH domain structure (Zheng, 2001). The PH (Pleckstrin homology) domain binds to phosphoinositides and thereby controls localization and the DH (Dbl homology) domain is responsible for catalyzing nucleotide exchange (Zheng, 2001). Dock7, a Dock180 related protein that catalyzes the nucleotide exchange of Rac1 (Watabe-Uchida et al., 2006), was found to specifically bind PIP_3_ via its DHR-1 domain (Kobayashi et al., 2001; Cote et al., 2005). Importantly, Dock7 is enriched in one of the stage 2 neurites—potentially the designated axon—supposedly controlling polarization and morphogenesis of the neuron (Watabe-Uchida et al., 2006). Controlled by these regulators, small GTPases can show phosphoinositide dependent activity patterns and a characteristic spatial distribution in the cell. However, direct evidence that GEF enrichment is a direct consequence of elevated PIP_3_ levels in a stage 2 neurite is largely missing. Accordingly, the spatial distribution of GEFs could also depend on another PIP_3_ binding protein which is recruited to and initiates a nascent axon.

As a result of their activation, GTP-bound GTPases engage in specific protein-protein interactions. By recruiting so-called effector proteins to the plasma membrane small GTPases determining the spatiotemporal activation pattern of other protein systems in the cell (Cherfils and Zeghouf, 2013). Effector proteins can be categorized in two classes: first, they can control cell morphology by directly acting on regulators of the actin or microtubule cytoskeleton, namely by increasing actin dynamicity and microtubule stabilization in the axon while stabilizing actin filaments in dendrites (Neukirchen and Bradke, 2011). For example, activation of Rac1 leads to a stabilization of axon microtubules via the stathmin pathway (Watabe-Uchida et al., 2006) while triggering actin remodeling (Hall et al., 2001; Gonzalez-Billault et al., 2012). In contrast, active RhoA promotes actin stabilization and contraction in dendrites as revealed by fluorescent activity sensors (Gonzalez-Billault et al., 2012). Second, effector proteins can be involved in the regulation of other small GTPases (DerMardirossian et al., 2004) or constituents of supramolecular complexes with various functions (Joberty et al., 2000; Lin et al., 2000). For example, they can act as coincidence detector for multiple binding partners (Carlton and Cullen, 2005) or signal to additional levels of regulation. For example, a common theme for the activation of small GTPases is that they comprise positive feedback loops. These self-amplifying circuits may not only lead to a local enrichment of GTPases on the membrane, but can also lead to collective, switch-like activation of proteins (Mizuno-Yamasaki et al., 2012). Accordingly, these kind of interactions can give rise to nonlinear signaling circuits with emergent properties, which can be crucial for breaking the symmetry and spatially organizing the cell (Yoshimura et al., 2006a).

The importance of positive feedback regulation for the symmetry breaking is probably best characterized in single-cell organisms such as yeast (Johnson et al., 2011). Despite the much lower complexity of this model organism, the general architecture of the biochemical network leading to cell polarization is most probably similar. In yeast, Cdc42 is the main spatial organizer of the cell as it regulates asymmetric cell division. Active, GTP-bound Cdc42 binds to the plasma membrane via its prenylated C-terminus, while GDP-bound Cdc42 is kept soluble in the cytoplasm via its interaction with its Guanosine nucleotide dissociation inhibitor (GDI) Rdi1. Cdc42-GTP is thought to form a locally confined protein cluster on the membrane by a local amplification of spontaneous asymmetries. The positive feedback is thought to arise from an effector-GEF complex, where the activated GTPase recruits an effector protein that in turn interacts with its activator. In yeast, a small, transient patch of Cdc42-GTP would recruit the scaffolding protein Bem-1 to the membrane, which interacts with the Cdc42 GEF Cdc24. Bem-1 not only binds to Cdc24 but also boosts its GEF activity. Thus Bem-1 efficiently catalyzes proximal Cdc42-GDP to exchange their nucleotide to Cdc42-GTP which again is able to recruit more Bem-1-Cdc24 complex (Nern and Arkowitz, 1998; Gulli et al., 2000; Johnson et al., 2011). Effector-GEF interactions have been found to be involved for the regulation of many different small GTPases (Mizuno-Yamasaki et al., 2012) and might be generally required for collective, switch-like activation of GTPases. These kind of decisive signaling reactions are of crucial importance for the cell, as they not only lead to cell polarization, but also regulate other fundamental processes such as membrane trafficking and the dynamic properties of the actin and microtubule cytoskeletons, thereby controlling the morphogenesis of the cell.

While the role of many proteins involved in neuronal polarity has been studied in neuronal cell culture, the function of Cdc42 has also been studied *in vivo* (Garvalov et al., 2007). Only about 30% of neurons derived from Cdc42 null mice were able to form a Tau-1 positive axon and the activity levels of the actin regulator cofilin were disturbed. However, when axon formation in Cdc42 null cells was initiated by cytochalasin, axons formed even if the drug was washed away. This indicates that Cdc42 is needed for the initial steps of axon specification but is dispensable for axon outgrowth (Garvalov et al., 2007).

### The PAR System

During cell polarization, the asymmetric distribution of phosphoinositides provides an initial signal to a number of protein systems that together control cell morphogenesis. One of those protein systems is the PAR system, which is recruited downstream of activated Cdc42 (Etienne-Manneville and Hall, 2001; Yamanaka et al., 2001; Nishimura et al., 2005). The PAR system is a set of highly conserved proteins that organize cell polarity in all metazoan cells. In neurons, it was found that the PAR system is required for axon dendrite polarity (Shi et al., 2003; Chen et al., 2006), migration (Sapir et al., 2008) and dendrite development (Terabayashi et al., 2007). However, a complete mechanistic characterization of how these proteins regulate axon formation is missing.

The PAR system is probably best studied in the nematode *Caenorhabditis elegans*, where it controls the first division. In a single cell embryo, the PAR proteins self-organize into two non-overlapping domains (anterior and posterior domain) to govern asymmetric spindle positioning and ultimately the generation of daughter cells with different fate (Kemphues et al., 1988; Gönczy and Rose, 2005). The PAR proteins have been categorized in anterior PARs (aPARs; PAR-3, PAR-6, PKC-3, cdc42) and posterior PARs (pPARs; PAR-2, PAR-1, LGL-1), all of which are peripheral membrane proteins. Their mutual exclusion is thought to arise from cross-phosphorylation by the two kinases PKC-3 and PAR-1, which leads to membrane detachment, controls oligomerization state and hence their diffusivity (Feng et al., 2007; Hoege and Hyman, 2013; Arata et al., 2016). PKC-3 can phosphorylate all posterior PARs (Hao et al., 2006; Hoege et al., 2010), in return, PAR-1 phosphorylates PAR-3 (Guo and Kemphues, 1995; Benton and St Johnston, 2003; Motegi et al., 2011). A network of regulatory biochemical interactions between aPARs and pPARs is thought to finely tune the activity of these kinases, leading to two dynamically stable cellular domains that govern a plethora of downstream events (Goehring, 2014).

Apart from PAR-2, the PAR system is conserved among most multicellular organisms and defines polarity via mutual exclusion in different contexts such as anterior-posterior polarity in *Drosophila* oocytes and apical-basal polarity in epithelial tissues (Morton et al., 2002; Goldstein and Macara, 2007; Thompson, 2013). The overall importance of the PAR system for axon dendrite polarity was firmly established in dissociated hippocampal cell culture system and enrichment of anterior PARs at the tip of the outgrowing axon has been observed (Shi et al., 2003). Subsequent knock down or overexpression studies of several PAR members showed that genetic manipulation of the PAR system either results in no or supernumerary axons (Figure 3). If mutual exclusion among aPARs and pPARs is a universal feature of the PAR system one would expect the posterior PAR-1 homolog MARK2 to be absent from the tip of the axon. Surprisingly, fluorescence sensors to measure MARK2 activity in the developing axon of cortical neurons showed highest kinase activity in the growing axon tip (Moravcevic et al., 2010; Timm et al., 2011). This indicates that both active PAR-1 kinases and aPARs are co-localizing at tip of the outgrowing axon and mutual exclusion of the “opposing” PAR complexes is not a requirement for axon dendrite polarity establishment. On a functional level, antagonism between MARK2 and the aPAR complex has been suggested (Chen et al., 2006). Analog to the *C. elegans* system, aPKC phosphorylates MARK2, which results in membrane detachment and most likely in reduced activity. Overexpression of MARK2 in hippocampal neurons prevented axon formation while knock down caused multiple axons (Chen et al., 2006; Wu et al., 2011). The opposite was seen for aPKC overexpression, which resulted in multiple axons (Figure 3; Parker et al., 2013). The overexpression phenotype of MARK2 was rescued by simultaneous overexpression of aPARs. No rescue was observed with a non phosphorylatable MARK2, indicating that direct inhibition of MARK2 by aPKC is responsible for the observed rescue. In a simple view, this could mean that MARK2 is a negative regulator of axon formation which is specifically inhibited by axon enriched aPARs via aPKC phosphorylation. However, *in vivo* knock out of the other PAR-1 homologs (SAD-kinases) also inhibited axon formation (see also above), indicating that a fine balance between these activities is needed (Kishi et al., 2005). Both, MARK2 and SAD kinases have to be activated by LKB1/PAR-4 (Lizcano et al., 2004; Shelly and Poo, 2011), which itself is downstream of cAMP/PKA signaling (Shelly et al., 2007). Thus, the PAR system not only translates PIP_3_ dependent signaling into altered cytoskeleton dynamics but also integrates the input of heterotrimeric G protein receptor ligands. Interestingly and in contrast to *C. elegans*, LKB1/PAR-4 is enriched in the axon (Shelly et al., 2007) while it is homogenously distributed in the *C. elegans* zygote (Goehring, 2014). Knockdown of LGL-1 prevents axon formation but the precise role of LGL-1 in axon development is still poorly understood (Plant et al., 2003; Wang T. et al., 2011).

Another fundamental difference between the neuronal and nematode PAR system is their dependence on a previous symmetry breaking event. In *C. elegans*, the sperm entry marks a single symmetry breaking event that starts actomyosin based flows and facilitates PAR domain establishment whereas no asymmetries of PIP_3_ have been reported. The PAR system in neurons is clearly downstream of regulators that directly control or are controlled by PIP_3_, such as PI3K or ATK/GSK3b, Cdc42 and Rap1B (Insolera et al., 2011), whereas initial polarity formation in *C. elegans* does not depend on these PIP_3_ controlled proteins (Schlesinger et al., 1999; Insolera et al., 2011). A possible explanation for this difference could be that neurons have to screen their environment during development (open systems) to remain a certain degree of plasticity while communication with the extracellular space is less important in the early stages of worm development. Thus, the PAR system is integrated into a more complex signaling network in neurons while it constitutes a rather autonomous polarity system in *C. elegans*. Since many PAR system intrinsic reactions (like phosphorylation events) seem to be conserved, it is still not clear how these reactions have to occur in space and time in neurons for faithful axon dendrite polarity establishment. Super resolution microscopy and higher temporal resolution of simultaneous activity monitoring of PARs and PIP_3_ may be required to solve the question of how PARs fulfil their functions during neuronal development.

### Closing the Loop

So far, we have only considered biochemical reactions downstream of an initial asymmetry of PIP_3_. For robust symmetry breaking, a self-enforcing loop is required, which would give rise to a local accumulation of PIP_3_ despite its rapid diffusion in the plasma membrane and the proteins in the cytoplasm. One possible functional network could originate from PIP_3_ and at the same time further increase its local concentration on the membrane. Therefore, the described functional modules need to talk to each other and eventually feed back to the activity of PI3K.

The molecular players involved for this regulatory network could for example be GTPases or their GEFs and effector proteins, which would not only translate local PIP_3_ enrichment into altered cytoskeleton dynamics and transport, but themselves further enhance the activity of PI3K. For example, Ras-GTP (Sasaki et al., 2004) and Rac1-GTP (Srinivasan et al., 2003) in combination with actin polymerization (Peyrollier et al., 2000; Wang et al., 2002) or via additional players like the Par6/Par3 aPKC complex where found to activate PI3K (Motegi et al., 2011; Laurin and Côté, 2014). Indeed, these proteins were all found to be required for axon formation (Shi et al., 2003; Yoshimura et al., 2006b; Tahirovic et al., 2010). H-Ras is a direct activator of PI3K and is also activated downstream of PI3K (Rodriguez-Viciana et al., 1994; Yang et al., 2012). Interestingly, this feedback loop results in H-Ras translocation via vesicle based transport into the future axon, which depletes H-RAS from the other neurites, presumably leading to reduced PI3K activity in other neurites and subsequently to inhibition of their outgrowth (Fivaz et al., 2008). While the idea of this amplifying circuit is at least partially based on experimentally verified protein-protein interactions, the emergent properties of this network have not been tested yet. For example, the role of PTEN localization and activity for phosphoinositide polarization in neurons is not yet clear (Kreis et al., 2014) and there might be functional networks that involve either less or a different set of molecular players. Furthermore, the connectivity of those circuits could even change with time, different extracellular inputs or in different subcellular locations.

Another layer of regulation can also be performed on the level of GDP dissociation inhibitors (GDIs), whose main function is to maintain their target, lipid-modified GTPases in an inactive, soluble state (Cherfils and Zeghouf, 2013). There is evidence that the affinity of RhoGDIs for different GTPases can be modulated by phosphorylation. For example, the kinase PAK1 is an effector protein of Rac1 that was found to phosphorylate RhoGDIs (DerMardirossian et al., 2004). Phosphorylation of these GDIs can enhance the dissociation of Rac1 from the GDI complex, thereby increasing the rate of Rac1 activation. As this leads to further stimulation of PAK1 activity such interaction may give rise to another positive feedback and symmetry breaking in neurons (Figure 2B).

Finally, and in addition to molecular processes that depend on locally confined phosphorylation and dephosphorylation of PIPs, PIP_3_ can also accumulate in the outgrowing axon with the help of directed microtubule-based transport. For example, the plus-end directed kinesin-like motor Gakin transports PIP_3_-containing vesicles through the interaction with the adaptor protein α-centaurin (Horiguchi et al., 2006). MARK2, a homolog of PAR-1, inhibits this transport by phosphorylating Gakin thereby preventing the development of axons (Yoshimura et al., 2010). MARK2 itself is deactivated by the PIP_3_-regulated kinase aPKC (Chen et al., 2006; Ivey et al., 2014). Thus a high local PIP_3_ concentration could inhibit MARK2 in the axon shaft, further enhancing directed transport of PIP_3_-containing vesicles to the growth cone. Accordingly, this could result in a self-perpetuating feedback loop supporting axon outgrowth (Yoshimura et al., 2010).

Collectively, these feedback loops stabilize polarity that can arise from short-lived local concentration fluctuations of external signals, temporal fluctuations in the output signal strength of receptors (Ladbury and Arold, 2012) or subtle heterogeneities on a coverslip. These small differences then lead to high and persistent activity of modulators that favor actin dynamicity and microtubule stability in the designated axon. Studies using drugs that either stabilized microtubules (Witte et al., 2008) or destabilized actin filaments (Bradke and Dotti, 1999) are sufficient to induce the formation of multiple axons, consistent with the view that the effects of the above mentioned circuits are transmitted via selective modulation of the cytoskeleton. In particular, these are the MARK2/SAD target and microtubule stabilizing tau proteins, microtubule destabilizers such as stathmin, actin dynamics modulators cofilin and WAVE and/ or inactivation of regulators that prevent axon outgrowth such as the RhoA/Rock module. This ultimately gives rise to a permanent molecular difference between axon and dendrites that will later on be manifested in a functional/electrophysiological difference of the two compartments, axon and dendrites. How this compartmentalization is maintained is not well understood and probably also relies on long range inhibitory signals, but future research will be needed to entangle the exact communications of these compartments during neuronal development.

## Conclusion and Perspectives

The phenomenon of neuronal polarization has been extensively studied in the last decades. Many of the analyses used the elegant cell culture system developed by Dotti et al. (1988). Thus the current model of neuronal polarization is to a large extent based on single hippocampal cells in an isolated system. Still, these extensive *in vitro* biochemical and cell biological analyses have provided a solid understanding of the general principles of cell polarization. A key question however remains: what are the cell-intrinsic biophysical and molecular mechanisms that induce the initial break in symmetry in cortical progenitor cells and developing cortical projection neurons *in vivo*? In order to address this question it will be essential to establish tools that allow the visualization and/or manipulation of the precise localization of molecular markers at high resolution in an *in situ* tissue context. The CRISPR-Cas9-dependent SLENDR method promises a high-throughput platform to visualize the endogenous localization of candidate proteins at high micro- to nanometer resolution (Mikuni et al., 2016). Given that a number of “polarity signaling systems” seem quite sensitive to perturbation and not particularly resilient, the precise determination of “polarity gene” function at distinct stages in development represents a current challenge in the field. In order to probe the function of genes encoding regulators of neuronal polarity *in vivo*, the genetic mosaic analysis with double markers (MADM) technology (Zong et al., 2005; Hippenmeyer et al., 2010; Hippenmeyer, 2013) offers an experimental opportunity. By exploiting MADM, one can induce sparse genetic mosaics with wild-type and mutant cells labeled in two distinct colors at high resolution. In combination with live-imaging such an experimental MADM paradigm enables: (1) the dissection of the cell-autonomous gene function; and (2) determination of the relative contribution of non-cell-autonomous effects *in situ* at the global tissue level (Beattie et al., 2017). Altogether, the experimental platforms above promise a robust approach to determine the so far unknown functions of regulators implicated in the polarization process of progenitor cells and nascent cortical projection neurons. A key open question in a functional context is: what is the level of redundancy and specificity in extracellular cues and intracellular amplification mechanisms? Interestingly, the process of MP-to-BP transition appears to involve not only dynamic cytoskeletal-associated processes but also regulation at the transcriptional level (Hippenmeyer, 2014; Ohtaka-Maruyama and Okado, 2015). It will be important to analyze transcriptional responses at high temporal resolution and evaluate the influence on the general biochemical cell state. In future experiments it will be also important to establish biochemical and biophysical methods and assays that should allow the precise analysis of the break in symmetry at high molecular and/or structural resolution. In a broader context it will be important to address the question whether cell-type diversity may imply the necessity for adaptation in the mechanisms controlling polarization? In other words, how conserved is the process of symmetry break and polarization in distinct classes of neurons with different morphologies? The future analysis of the core signaling modules controlling cell polarity in a variety of brain areas and at high cellular and molecular resolution promises great conceptual advance.

## Author Contributions

AHH, CD, CM, ML and SH contributed equally to the writing of the initial draft. All authors revised the manuscript.

## Conflict of Interest Statement

The authors declare that the research was conducted in the absence of any commercial or financial relationships that could be construed as a potential conflict of interest.
